# Clinical analysis of neuromyelitis optica spectrum disease with area postrema syndrome as the initial symptom

**DOI:** 10.1186/s40001-022-00949-9

**Published:** 2022-12-29

**Authors:** Ting Liu, Lijuan Li, Xiaopeng Guo, Qifu Li, Dandan Jia, Lin Ma

**Affiliations:** Department of Neurology, The First Affiliated Hospital of Hainan Medical University, Haikou, Hainan China

**Keywords:** Neuromyelitis optica spectrum disorders, Area postrema syndrome, Magnetic resonance imaging, Biomarkers

## Abstract

**Objective:**

The objective of this study was to report and discuss clinical analysis, including the diagnosis and treatment of 4 cases of neuromyelitis optica spectrum disease (NMOSD) with area postrema syndrome (APS) as the first symptom.

**Methods:**

Four patients with intractable nausea, vomiting, and confirmed NMOSD were included in the final analysis. All of these patients were initially misdiagnosed and mismanaged.

**Results:**

Among the 4 patients, 3 were admitted to the department of gastroenterology at the onset of the disease, and 2 were not correctly diagnosed and treated promptly due to misdiagnosis. Therefore, their symptoms worsened, and they were transferred to Intensive Care Unit (ICU) for life support. No obvious early medulla lesions were found in one patient. One patient was treated with intravenous immunoglobulin, methylprednisolone, and plasma exchange, but there was no significant clinical improvement, after which the disease relapsed during the treatment with low-dose rituximab.

**Conclusion:**

The clinical manifestations of NMOSD are complex and diverse, and the initial symptoms, onset age of the patient, and magnetic resonance imaging (MRI) findings can influence the final diagnosis. Early identification of the APS and timely therapy can prevent visual and physical disabilities, even respiratory failure, coma, and cardiac arrest. Therefore, it is necessary to identify specific and sensitive serum and imaging markers for predicting the prognosis and recurrence of the disease.

## Introduction

Neuromyelitis optic spectrum disease (NMOSD) is an immune-mediated inflammatory demyelinating disease of the central nervous system. The typical clinical manifestations include optic neuritis (ON) and longitudinally extensive transverse myelitis (LETM). Area postrema syndrome (APS) is relatively rare among the clinical symptoms of NMOSD and often presents as nausea and vomiting of unknown cause, which is clinically easily misdiagnosed. According to available data, the incidence of isolated APS in NMOSD patients in the UK, Japan, and the US is 7.1%, 8.7%, and 10.3%, while the incidence of APS with other symptoms at the time of onset (< 30 days interval) was 11.2%, 15.9%, and 8.2%, respectively [1]. In this study, we retrospectively analyzed the clinical data of 4 patients with NMOSD with the onset of APS and discussed the pathogenesis, clinical characteristics, diagnosis, and treatment of NMOSD to improve the understanding of this disease and reduce misdiagnosis and mistreatment of patients.

## Method

### Study population

The current 4 cases were selected among the 54 patients with AQP4-IgG-positive NMOSD who were diagnosed in our hospital between 2018 and 2021. Initially, there were 5 patients presenting with APS, but 1 patient was excluded due to incomplete medical records. Four patients included in this study met the 2015 international diagnostic criteria [2]. These diagnoses were made independently by three experienced neurologists. All 4 patients agreed to the publication of their anonymized clinical data.

### Data collection

The patients' medical records were reviewed, and demographic, clinical, laboratory, and MRI data were collected. Information on intractable nausea, vomiting, or hiccups (INVH) duration, department of the first visit, the time interval from INVH to the development of core NMOSD syndrome, and other core NMOSD features, such as acute diencephalon, acute brainstem, symptomatic brain syndrome, and optic neuritis and acute myelitis, were also collected.

## Results

### Case records

**Fig. 1:**
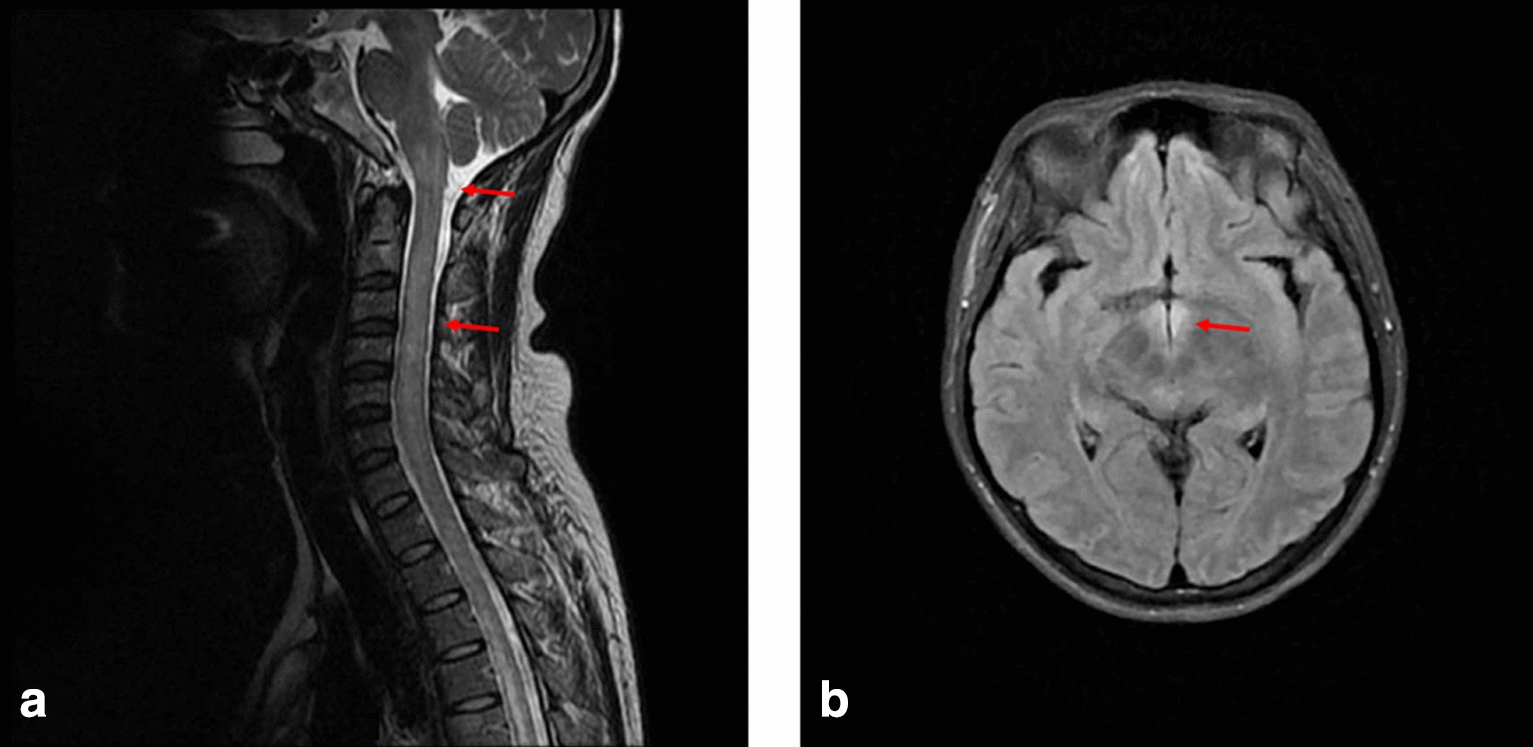
MRI T2 sequence of case 1 showing hyperintense in the medulla oblongata, cervical medulla, and thoracic medulla (highlighted by arrows) in the **a** sagittal planes, and hyperintense in the bilateral hypothalamus in the **b** transverse planes

Case 1 was a 31-year-old female patient who developed nausea and vomiting of unknown cause more than one month before admission. She was hospitalized in three hospitals and was diagnosed with ‘‘reflux esophagitis and chronic gastritis’’ following gastroscopy; however, the antiemetic treatment was ineffective. Fever and delirium occurred during this period. After taking antipsychotics for 4 days before admission, she suffered acute impairment of consciousness, lasting more than 10 h. After waking up, she felt tetraparetic, and her inability to achieve orthostatic position, dyspnea, and difficulties with urination and defecation. After being transferred to ICU at our hospital, an MRI plain scan of the brain and neck showed multiple abnormal hyperintense in the spinal cord from the medulla oblongata to the L1 level and scattered abnormal signals in the bilateral hypothalamus, dorsal pons, and medulla oblongata (Fig. 1). The serum AQP4-IgG was positive for the first time, and the patient was diagnosed with NMOSD. She accepted intravenous methylprednisolone (IVMP) and was treated with intravenous immunoglobulin (IVIg) after the reduction of the first dose, which was followed by 3 plasma exchanges. One month after admission, the patient received continuous 4 weeks of low-dose rituximab treatment. Before the end of the treatment course, her limb weakness worsened and was accompanied by nausea, blurred vision, and diplopia. Relapse was considered, and IVIg was given to improve the condition and discharge.

**Fig. 2:**
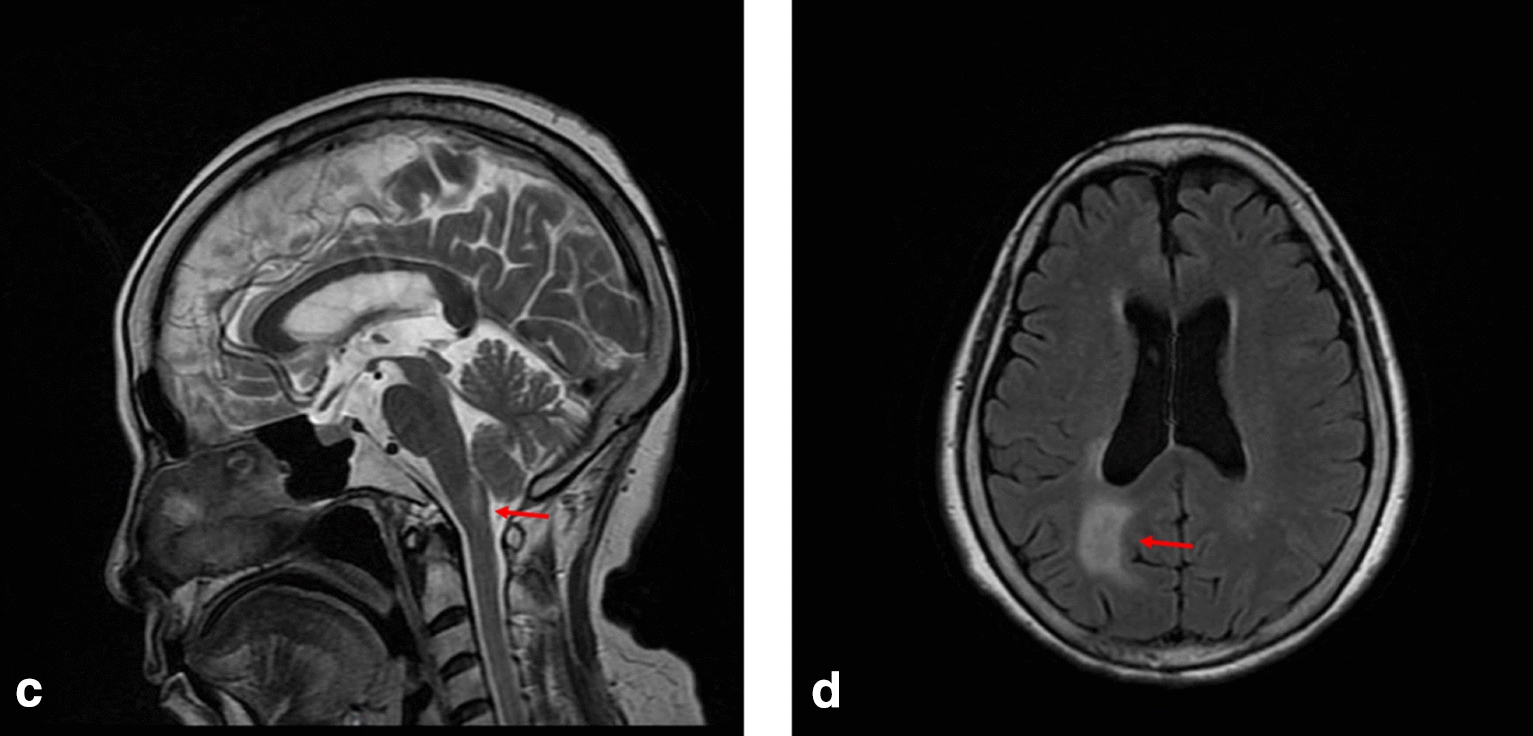
MRI T2 sequence of case 2 showing hyperintense in medulla oblongata (highlighted by arrows) in the **c** sagittal planes, and hyperintense in the right paraventricular and left occipital lobe in the **d** transverse planes

Case 2 was a 62-year-old female patient who developed nausea, vomiting, numbness, weakness in the left limb, dizziness, and headache 1 month ago after catching a cold. She came to our hospital and in the follow day and MRI was performed, MRI showed abnormal hyperintense in the right thalamus, paraventricular region, and left occipital lobe (Fig. 2). Physical examination showed 4-grade muscle strength in the left limbs. She was diagnosed with cerebral infarction and discharged after treatment. One month later, the patient returned to the hospital due to dizziness and headache. MRI showed that the original lesion was larger than before, but oligoclonal bands were negative, and the patient was discharged from the hospital. Subsequently, she searched medical care twice due to recurrent nausea and vomiting. Physical examination again showed mild hemiparesis on the left (MRC grade 4). Considering the possible presence of APS during this time, the improved AQP4-IgG test in cerebrospinal fluid was found to be positive, and the patient was diagnosed with NMOSD. Again, she was discharged after accepting IVMP. She has been taking mycophenolate mofetil ever since. At present, she has a good recovery of limb muscle strength, can walk independently, and has no obstacles in daily life.

**Fig. 3:**
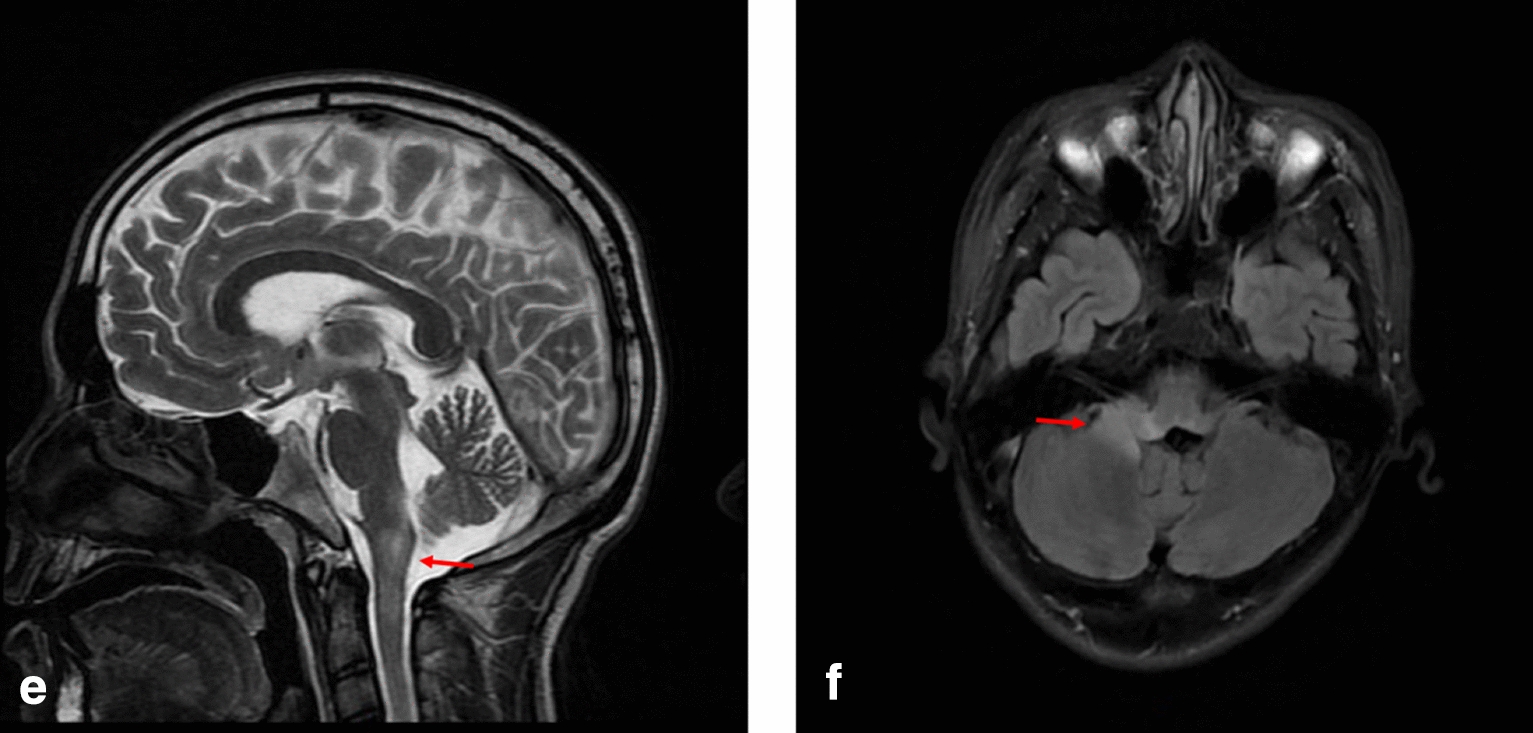
MRI T2 sequence of case 3 showing hyperintense in medulla oblongata (highlighted by arrows) in the **e** sagittal planes, and hyperintense in the right cerebellum and brainstem in the **f** transverse planes

Case 3 was a 23-year-old male who presented with intractable nausea and vomiting 1 month ago and was discharged from the hospital after treatment for chronic gastritis. One month later, the patient went to the department of gastroenterology again due to nausea and vomiting. Three days after admission, his nausea and vomiting worsened and were accompanied by limb weakness, dizziness, headache, and ataxia. Physical examination revealed bilateral nystagmus, occasional dysarthria, dysphagia, unsteady walking, and dysmetria on the upper right limb. MRI of the cervical spine and thoracic spine showed multiple abnormal hyperintense changes in the medulla oblongata, upper cervical medulla (C1-2 vertebral level), and thoracic medulla (T1-2 vertebral level) (Fig. 3). The patient was transferred to the department of neurology as neurological diseases were considered. Improved AQP4-IgG test in cerebrospinal fluid was found to be positive, and the patient was diagnosed with NMOSD. Three days later, the patient suddenly developed a disturbance of consciousness, decreased oxyhemoglobin saturation, and decreased blood pressure. He was immediately given an oral trachea cannula and breathing machine-assisted breathing, after which he was transferred to ICU for treatment. He was discharged after accepting IVMP and IVIg. Two years later, the patient was admitted with diplopia, and a brain MRI showed a medulla oblongata lesion extending to the right cerebellum. Disease relapse was considered, and after accepting IVMP, the patient was discharged after symptom improvement.

**Fig. 4:**
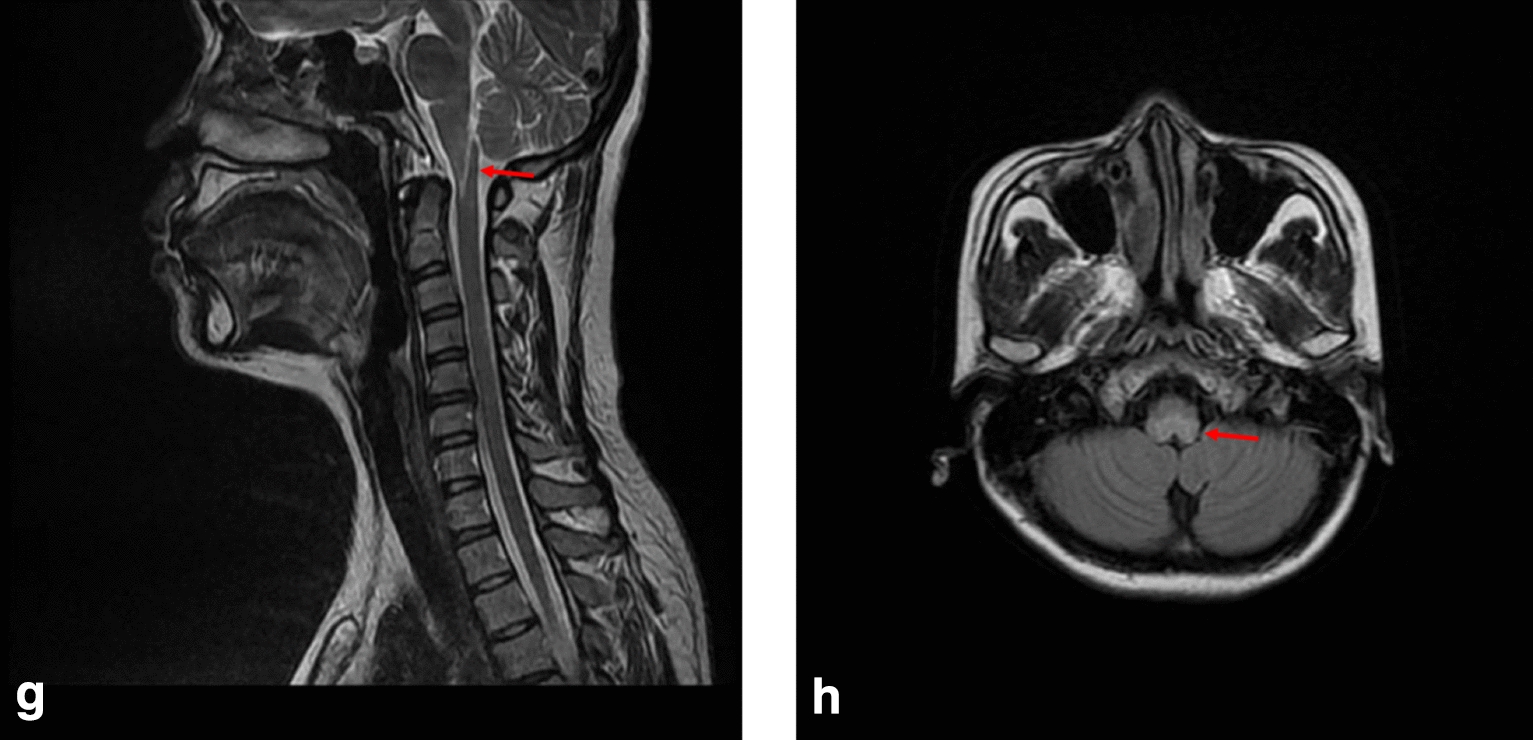
MRI T2 sequence of case 4 showing hyperintense in medulla oblongata (highlighted by arrows) in the **g** sagittal planes and **h** transverse planes

Case 4 was a 30-year-old woman who was diagnosed with reflux esophagitis in the department of gastroenterology at another hospital after repeated vomiting for 2 months, with no positive signs on neurological examination. Two months later, the patient was admitted to the neurology department due to numbness and weakness in the right limb. Physical examination revealed tetraparesis (the UK Medical Research Council, MRC grade 4) and shallow left nasolabial fold. MRI of the cervical spine demonstrated abnormal hyperintense at the medulla oblongata to the C2 level (Fig. 4). The possibility of central nervous system demyelination disease was considered, and the patient was treated with IVMP and discharged. Three years later, she reappeared in tetraparesis. AQP4-IgG test in serum was positive. She was discharged after accepting IVMP. The patient has been taking mycophenolate mofetil ever since.

### Patient demographics and clinical characteristics

Among the above 4 cases, 3 were female and 1 was male. The onset age ranged from 23 to 62 years, with an average onset age of 36.5 years. All patients developed intractable nausea, vomiting, or hiccups; these symptoms were excluded from the combination of other systemic diseases. Three patients had acute myelitis, 2 had optic neuritis, 3 had acute brainstem syndrome, 2 patients had the acute diencephalic syndrome, and 2 patients had cerebral syndrome. All 4 patients had typical relapses, and 2 of them had persistent nausea, vomiting, or hiccups.

### Laboratory findings

Laboratory test data of patients are summarized in Tables 1, 2. Serum AQP4-IgG was positive in all 4 patients. Two of the three patients tested for AQP4-IgG in CSF were positive. Recurrent hyponatremia occurred in 2 patients, thyroid peroxidase antibody was elevated in all patients, and rheumatoid factor was positive in 2 patients. In the antinuclear antibody test of 4 patients, 2 patients were positive for anti-SSA antibody and anti-Ro52 antibody. pANCA-IgG antibodies were found in 1 patient.

**Table 1:** Laboratory findings of APS-NMSOD patients.

*n*	sex	First diagnosis	Duration of INVH(d)	Interval time from APS to OCS (d)	CSF leucocyte count (×10^6^/L)	TP(mg/L)	Cl (mmol/l)	Lactate dehydrogenase(U/L)	Electrolyte disturbance	Other Ab
1	F	Reflux esophagitis	25	25	225	975.56	120	64.94	hyponatremia	SSA;Ro52;RA
2	F	Cerebral infarction	1238	29	3	287.4	117.5	21	hyponatremia	–
3	M	Chronic gastritis	37	834	–	257	116.6	39	–	SSA;Ro52;RA
4	F	Reflux esophagitis	65	69	5	433.4	121.2	29	–	pANCA

*N* number, *Ab* antibody coexisting, *OCS* other core symptoms

**Table 2:** MRI findings and AQP4-Ab/OB of APS-NMSOD patients

*n*	Lesion	AQP4-Ab	MOG-Ab	OB
1	MO; Bilateral hypothalamus; VT; DP; C2-T1	+	–	–
2	MO; right thalamus; periventricular; VT; left occipital lobe	+		–
3	MO; C1-2; T1-2	+	–	–
3	MO; C1-C2; left occipital lobe	+	–	–

*MO* medulla oblongata, *VT* ventriculus tertius, *DP* dorsal pontine

### MRI findings

The MRI findings of the 4 patients in the study are summarized in Table 2. MRI of all patients showed abnormal signals of the dorsolateral medulla oblongata, no noticeable medulla lesions earlier in 1 patient, and 2 patients with medulla oblongata lesions connecting to the cervical spinal cord. During follow-up, progression of cerebellar lesions to the medulla oblongata was observed in 1 patient. MRI showed abnormal signals in the diencephalon in 2 patients and abnormal signals in the cerebral hemisphere in 2 patients.

### Therapy and EDSS score

The therapy and EDSS scores of the 4 patients in the study are summarized in Table 3.

**Table 3:** Therapy and EDSS score before treatment

*n*	Acute attacks	IST	EDSS score before treatment
1	IVMP; PE; IVIg	Rituximab	8.0
2	IVMP	Mycophenolate mofetil	2.5
3	IVMP; IVIg	–	1.5
4	IVMP	Mycophenolate mofetil	2.0

*IST* immunosuppressive therapy, *IVMP* intravenous methylprednisolone, *IVIg* intravenous immunoglobulin, *PE* plasma exchange

## Discussion

Current epidemiological survey data suggest that the first onset of NMOSD mostly occurs in young and middle-aged people, predominantly women, having a higher incidence in African, Asian, and Latin American populations than in white populations. Due to the small sample size and different AQP4 antibody detection techniques, the incidence of NMOSD in different regions is still controversial. It is also worth mentioning that with evolution of detection technology and more understanding of the pathogenesis of the disease, the incidence of the disease has been globally increasing on a yearly basis [3–6]. One study reported that in 100 patients with APS as the initial presentation of NMOSD, women were more commonly affected. Also, their epidemiological data were similar to other NMOSD patients [1]. NMOSD has a high recurrence rate and disability rate in the course of the disease, but the pathogenesis and recurrence factors are not completely clear. Multi-center studies have shown that NMOSD is related to genetics, environment, autoimmunity, infection, climate, and other factors [7, 8].

In addition to the common optic neuritis and acute myelitis, patients with APS often experience missed diagnosis and misdiagnosis. APS can appear as an isolated clinical symptom in the early stage of the disease, often presenting as intractable nausea and vomiting. Therefore, APS is often encountered in other departments besides neurology [9]. Among the 4 patients included in the present study, 3 were admitted to the department of gastroenterology at the onset of the disease, and 2 of them were not correctly diagnosed or timely treated due to misdiagnosis, so their symptoms worsened, and they needed to be transferred to ICU for life support. Therefore, it is of great importance to improve understanding of the disease. The pathogenesis of APS is mainly related to aquaporin 4 antibody (AQP4-IgG). AQP4 is highly expressed in certain areas of the nervous system, such as the thalamus, hypothalamus, corpus callosum, periventricular, spinal cord, optic nerve, and area postrema. The area postrema serves as nausea, vomiting, and hiccup center, and vomiting-related chemical stimuli can cause nausea and vomiting by stimulating chemoreceptors in the area postrema [10, 11]. In the present study, case 2 was misdiagnosed with cerebral infarction and failed to receive a correct diagnosis and timely treatment for an extended period of time. A multi-center retrospective study in South Korea divided patients into early-onset NMOSD (EONMOSD, age of first onset ≤ 50 years) and late-onset NMOSD (LONMOSD) according to their age of onset, finding that the age of onset was an important factor affecting the course of the disease and clinical prognosis. Moreover, 62.2% of LONMOSD patients presented with transverse myelitis, and 37.2% presented with optic neuritis. Other clinical manifestations were rare [12]. Another study also supported this view, as LONMOSD has fewer lesions around the fourth ventricle and more in the cerebral hemisphere than EONMOSD [13]. By comparing 12 male LONMOSD patients and 64 female LONMOSD patients, other scholars found that the onset of transverse myelitis was less common in males, and the time from onset to diagnosis was shorter compared to females. Also, their EDSS score was lower [14]. Clinical diagnosis of NMOSD is often confused by gender, age of onset, and atypical clinical manifestations. Some patients may have monoplegia or hemiplegia due to different lesion locations, which needs to be differentiated from ischemic cerebrovascular disease.

In order to improve the diagnosis rate of APS, experts in the region in the region and internationally have reached a consensus on the diagnostic criteria of APS. Clinical acute or subacute intractable nausea, vomiting and/or hiccup, and symptoms lasting more than 48 h should indicate APS after exclusion of other causes, and the diagnosis can be confirmed after the improvement of the AQP4 antibody test [1, 2]. In recent years, scholars found that compared with AQP4-Ab-seropositive patients, double-seronegative and myelin oligodendrocytes glycoprotein (MOG)-Ab-seropositive patients had less severe clinical attacks and better prognoses, including lower EDSS scores and a lower proportion of disability [15, 16]. This is consistent with our data.

In humans, AP is a V-shaped structure consisting of two short, thick branches that are located at the base of the fourth ventricle, which connects to the midline of the most caudal medulla. A previous study found that the "inverted V sign" in the medulla oblongata on axial MRI T2 FLAIR sequence in patients with APS has high specificity for diagnosis. In addition, sagittal MRI in most APS patients also shows "linear sign," which is also seen in other diseases besides NMOSD [17, 18]. In the present study, case 2 underwent cranial MRI procedures several times before the diagnosis, but no obvious lesions on the dorsolateral medulla oblongata were identified. These observations caution clinicians to consider the central nervous system demyelinating diseases for patients with intractable nausea and vomiting without apparent medulla oblongata lesions on the imaging.

NMOSD patients are often associated with other autoimmune diseases, such as systemic lupus erythematosus and Sjogren's syndrome [19]. Previous studies have found thymus degeneration in NMOSD patients, which may be an important cause of systemic immune dysfunction [20]. The positive antinuclear antibody was not significantly correlated with the course and severity of NMOSD; however, the positive antinuclear antibody was more likely to be accompanied by the increase of other antibodies than the negative antinuclear antibody [21]. So far, no large sample study has demonstrated the existence of specific autoimmune diseases among NMOSD patients with different clinical symptom onset.

The symptoms of most NMOSD patients improve after high-dose steroids (HDS) treatment [14], but a small number of patients are still insensitive to HDS. Patients who are not responding to HDS therapy during acute attacks may be treated with intravenous immunoglobulin (IVIg), which reduces disability and prolongates the time to relapse [22]. In addition to IVIg, immune adsorption (IA) and plasma exchange (PE) have been suggested to be equally effective in the treatment of acute attacks [23]. Compared with IVMP alone, HDS combined with IVIg was found to be superior to IVMP alone. Comparing the data of 243 patients with acute onset NMOSD, it was concluded that HDS combined with IVIg was superior to HDS alone in patients with EDSS > 6. However, continued use of IVIg does not improve the outcome of patients failing to respond to HDS treatment [24], which might explain why case 1 in this study received IVIg and PE after HDS without significant clinical improvement.

Rituximab (RTX) is increasingly used in the treatment of NMOSD by depleting CD20 + B cells, thereby suppressing the immune system. Several studies have confirmed that RTX's safety and effectiveness, and the low incidence of adverse drug safety events and opportunistic infections are more advantageous than other therapeutic drugs [25–27]. In China, low-dose RTX is mostly used to prevent the recurrence of NMOSD. RTX can significantly reduce the recurrence rate of NMOSD and effectively improve the degree of disability of patients; yet, there are still a small number of patients with short-term recurrence after RTX [28, 29]. Some scholars believe that it takes time for RTX to eliminate pathogenic antibodies and that peripheral B cells are mainly affected, while a single course of RTX treatment cannot eliminate specific memory B cells [30, 31]. In our study, B cells were not monitored during the application of low-dose RTX only in case 1, so the dosage of RTX could not be adjusted, which may be one of the reasons for the short-term recurrence of the disease in the patient. Adjusting the dose of RTX treatment according to the number of B cells and individualizing the treatment plan could potentially reduce the short-term recurrence of RTX treatment in the future.

Older age, the shorter time between onset and subsequent relapse, and lack of immunosuppressive therapy (IST) have been associated with higher mortality, regardless of the severity of the onset presentation [32]. In the follow-up of 17 patients with NMOSD, 14 of them relapsed after six months of drug withdrawal, suggesting that in patients with severe clinical symptoms, IST treatment should be carefully discontinued [33]. A Chinese single-center study also supports the view that more caution is needed when discontinuing IST, regardless of the duration of use [34]. Due to the existence of respiratory and cardiovascular centers in the upper cervical spinal cord and lower brain stem, the progression of medulla oblongata lesions in NMOSD patients is likely to endanger patients' life. Therefore, patients with NMOSD with APS as an initial symptom should undergo careful clinical evaluation before careful discontinuation of drugs.

Clinically assessing whether a patient with NMOSD needs to stop treatment, intensify treatment, or initiate preventive treatment is difficult due to the lack of markers that can be used to predict the disease. It was previously found that serum glial fibrillary acidic protein (sGFAP) was slightly higher in patients with NMOSD in clinical remission compared with healthy controls, and sGFAP was significantly higher in acute attacks compared with remission. SGFAP may be used as a biomarker of disease activity in remission in the future. Higher sNfL levels were also found to indicate a shorter onset time [35]. A prospective study in China suggested that sNfL could be used as a biomarker for the severity of NMOSD, and higher levels of TH-2-related cytokines (by antagonizing IL-1, IL-4, IL10, and IL-13 receptors) were involved in the process of disease remission [36]. In addition, IL-6, IL-10, C3, and C4 are thought to be involved in the occurrence of diseases and may become potential biomarkers for clinical diagnosis and treatment [23, 37–39].

## Conclusion

The clinical manifestations of NMOSD are complex and diverse, and the initial symptoms, onset age, and MRI can all affect the diagnosis of the disease. Early identification of APS and timely therapy can prevent visual and physical disabilities, even respiratory failure, coma, and cardiac arrest. Withdrawal of clinical drugs in NMOSD patients needs to be evaluated from several different aspects. The treatment course can be appropriately extended for patients with older onset and APS to reduce disease recurrence. It is necessary to strengthen the study of serum and imaging markers to predict the disease's progression and recurrence.

## Data Availability

Data supporting these findings are available from the corresponding author upon request. The data cannot be made publicly available because of privacy or ethical restrictions.
